# Optimizing Dental Implant Success: Biomechanical Insights, Computer-Guided Precision and The Role of Open Guide Systems in Achieving Cost-Effective and Accessible Treatment

**DOI:** 10.12669/pjms.41.9.11808

**Published:** 2025-09

**Authors:** Abdulaziz Abdullah Alkhureif, Majed M. Alsarani, Aftab Ahmed Khan, Aisha Wasi

**Affiliations:** 1 Abdulaziz Abdullah Alkhureif, PhD, MPhil, BDentTech Professor, Dental Health Department, College of Applied Medical Sciences, King Saud University, Riyadh, Saudi Arabia; 2 Majed M. Alsarani, PhD, MSc, BDentTech Associate Professor, Dental Health Department, College of Applied Medical Sciences, King Saud University, Riyadh, Saudi Arabia; 3 Aftab Ahmed Khan, PhD, MSc, M.Bioeth, BDS Researcher, Dental Health Department, College of Applied Medical Sciences, King Saud University, Riyadh, Saudi Arabia; 4 Aisha Wasi, BDS Demonstrator, Dental Health Department, College of Applied Medical Sciences, King Saud University, Riyadh, Saudi Arabia

**Keywords:** Computer-assisted dental implant surgery, Dental implants, Placement of implant, Vision measuring machine

## Abstract

**Objective:**

This study aimed to compare the positional and angular accuracy of digitally planned implant placements with an open-guide system, using a vision measuring machine (VMM).

**Methodology:**

This in vitro experimental study, conducted at King Saud University (May to September 2024), involved placing 24 endosseous implants into eight replaceable bone blocks. Three implants were placed in each bone block (Nissin™ Dental, Japan), measuring approximately 50 mm (L) × 25 mm (W) × 20 mm (H). A jaw model simulating a partially edentulous mandible was used. Preoperative CBCT scans and virtual surgical planning software guided the fabrication of stereolithographic surgical templates. Post-placement, implant accuracy was assessed using the VMM.

**Results:**

The open-guide system (Group-B) demonstrated significantly improved linear (e.g., L I– II: 1.09 mm, p = 0.001) and angular accuracy (e.g., P III deviation from 90º: 9.59º, p = 0.000) compared to digital planning alone (Group-A). Vertical accuracy, however, was inconsistent, with some deviations reaching significance (e.g., V DI: p = 0.000), while others did not (e.g., V DII: p = 0.552).

**Conclusion:**

The stereolithographic open-guide system enhances linear and angular precision in implant placement, particularly in multi-implant cases. However, vertical accuracy remains less predictable, warranting further refinement for improved surgical outcomes.

## INTRODUCTION

Over the past three decades, implant-supported prostheses for fully edentulous patients have evolved from a contentious option to a well-established, highly predictable treatment modality.^1^ Implant dentistry, while protocol-driven, remains a dynamic field emphasizing meticulous planning for the rehabilitation of missing teeth.^2^ This paradigm shift reflects a dual focus: addressing bone deficiencies and strategically designing implant positions based on the intended final prosthetic outcome.

Functional and esthetic complications are possible results of malpositioned implants and this underscores the need for accuracy during placement.^3^,^4^ Guided implant surgery, particularly using image-guided techniques has been crucial in achieving the best outcomes.^5^ It merges cone beam computed tomography (CBCT) with computerized planning tools for virtual implant placement to enhance both form and function. Image-guided surgery integrates preoperative diagnostic imaging, such as CBCT, with computer-based planning tools to ensure precise surgical and restorative outcomes.^6^

CBCT has revolutionized treatment planning in implant dentistry by offering high resolution and a three-dimensional visualization of all maxillofacial structures. Nevertheless, CBCT is limited by reduced soft tissue contrast, susceptibility to motion artifacts and image distortion from metallic objects.^7^ In contrast, the Vision Measuring Machine (VMM), a non-contact optical instrument, provides sub-millimetric dimensional accuracy for assessing mechanical deviations in implant positioning in vitro. VMM enables reproducible assessment of linear and angular deviations between planned and actual implant positions. Nonetheless, its application is restricted to ex vivo studies due to the requirement for fixed specimens and a controlled environment, limiting its clinical use.^7^,^8^

Computer-generated surgical guides have significantly enhanced the accuracy and efficiency of implant dentistry by offering customized solutions for precise implant placement across diverse edentulous conditions.^9^ These guides are classified as tooth-supported, mucosa-supported, or bone-supported based on the patient’s edentulous status. Tooth-supported guides are employed in partial edentulism cases, mucosa-supported guides are used for fully edentulous patients, relying on accurate jaw relations and fixation with screws or pins, while bone-supported guides are primarily utilized in fully edentulous patients with severe ridge atrophy where mucosa-supported guides cannot be reliably seated.^10^ Through stereolithography, custom surgical guides can be manufactured, allowing precise control of implant depth, angulation and positioning.^11^,^12^ Multiple studies have compared the accuracy of implant positioning using mucosa-supported versus bone-supported surgical guide templates for different guide types in the context of mandibular complete implant-supported overdentures.^10^,^12^

The rising demand for dental implants driven by economic factors underscores the need for cost-effective, efficient solutions.^13^ This study evaluates discrepancies between planned and actual implant positions to enhance accuracy in 3D image-guided implant rehabilitation. High-precision guided placement improves surgical efficiency, reduces iatrogenic trauma and ensures predictable outcomes, thereby optimizing resources across diverse clinical settings.^7^ To the best of our knowledge, this study is among the first to comprehensively evaluate and directly compare the clinical accuracy of a universally adaptable open-guide surgical system with CBCT-based virtual implant planning, using high-precision VMM analysis. While previous studies have explored guided implant placement, few have employed micrometric tools like VMM to objectively quantify linear and angular deviations from planned positions. By integrating advanced imaging for planning and VMM for outcome validation, this study introduces a novel methodological framework that enhances understanding of the accuracy and clinical potential of open-guide systems in implant dentistry.

## METHODOLOGY

This study was conducted at the Dental Health Department, King Saud University Riyadh, Saudi Arabia between May to September 2024. The study materials are listed in Table-I. Mandibular posterior edentulous bone-like replaceable bones (NissinTM dental, Japan) were fabricated for placement of twenty-four endosseous implants (Adin Touareg-S, Adin Implant System Ltd., Israel). Bone blocks (Nissin™ Dental, Japan) were pre-fabricated polyurethane models simulating type I dense bone, manufactured using high-pressure molding to replicate human mandibular posterior segments. A total of eight bone blocks were used in the study. Three implants were placed into each block. The accuracy of the 24 implants was then evaluated based on. linear measurements (mesiodistal and vertical) and angular measurements (n=24). The baseline (control) was the planning derived from the CBCT method for placing three implants in one bone block.

**Table-I:** Materials used in this laboratory study.

Material	Manufacturer	LOT/ BATCH NO.
Partially edentulous jaw model	Nissin™ Dental, Japan	IMP1001-UL-SP-FEM
Light Cured custom tray material	ELITE LC tray, Zhermack Spa, Italy	D500021
Gutta Percha Points	DIADENT^®^, Vancouver, BC Canada	010315
Stereolithographic Open Surgical Template/Guide	In 2 GuideTM, Cybermed, Seoul, South Korea	----
Self-Cure Acrylic Resin	Travelon, Dentsply, USA	LP-0415
Polyether impression material	Monophase; 3M ESPE™, St Paul, Minn, USA	608553
Pentamix™ 2	3M ESPE™, St Paul, Minn, USA	----
Type IV die stone	Ultrarock, Kalabhai Karson Pvt Ltd, Mumbai, India	160708
In 2 GuideTM Surgical Template	KaVo Dental GmbH, Seoul, South Korea	----
Adin Touareg-S, two-piece, endosseous implants	Adin Implant System Ltd. Israel	----

### Ethical Approval:

Exemption from the Research Ethics Committee of the College of Applied Medical Sciences was obtained Ref. E-004-3738, Date: April 23, 2024).

A jaw model was articulated to simulate a completely dentulous maxilla (Nissin™ dental, Japan) to get the occlusion of replaced teeth accurately. A radiographic template was made that mirrored the intended prosthetic result. Upper and lower jaw models were created using monophase polyether imprints (3M™ ESPE, USA) and duplicate castings were made using Ultrarock type IV dental stone. A radiographic stent was created on the mandibular cast and a specially designed tray was light-cured for 10 minutes. Each of the template buccal and lingual flange surfaces had several openings made on them with a diameter of 1mm and distributed at different elevations below the cervical margins and relative to the occlusal plane. To locate the implants, various radiographic markers containing gutta-percha balls (DIADENT^®^, Diadent Group International Inc., Vancouver, BC Canada) were inserted into these perforations.

CS 9300 Cone Beam CT Unit (Carestream Dental Systems, Carestream Health Inc, Rochester, NY, USA) was used to obtain cone beam scan images of the mandibular model with a radiographic stent. The exposure parameters were set at 85-90 kV and 10-12 mA. There was also a second scan following the Dual scan protocol. The software (OnDemand3D™, Cybermed Inc., South Korea) used radiopaque markers in the radiographic template to merge two scans, creating a precise 3D model of the bone and template. CBCT-generated DICOM data was used by implant planning software (In2Guide™, Cybermed Inc.) for virtual implant planning in 2D and 3D, aligning the segmented 3D cast and radiographic guide through spherical markers. The software selected appropriate dental implants (Adin Implant Systems Ltd., USA) and facilitated virtual surgical planning, determining implant location, angle and depth.

The completed simulation was saved as an STL file for stereolithographic fabrication of the surgical guide. The guide was created using stereolithographic equipment (In2Guide™, Cybermed Inc.), targeting three implant locations (35, 36, 37) with specific sizes: 3.75 × 10 mm (35) and 4.2 × 10 mm (36 & 37). Planned inter-implant distances were 8.71 mm, 8.79 mm and 17.50 mm. Implant sites were planned supracrestal at 1.5 mm from the ridge crest and vertical and angular measurements were determined using CBCT data (Table-II). These inter-implant distances were based on anatomical measures to ensure adequate inter-implant spacing and accommodate future prosthetics. The supracrestal positioning at 1.5 mm was chosen to mimic clinical recommendations for biologic width preservation and ease of surgical access.

**Table-II:** Vertical and Angular Measurements of Implants planned on CBCT.

Implant Location	Vertical Measurement	Angular Measurements
Implant placed in 35 location (mesial)	1.51 mm	90º
Implant placed in 35 location (distal)	3.37 mm	
Implant placed in 36 location (mesial)	1.51 mm	90º
Implant placed in 36 location (distal)	1.51 mm	
Implant placed in 37 location (mesial)	2.47 mm	90.1º
Implant placed in 37 location (distal)	1.51 mm	

A stereolithographic guide was created using a rapid prototyping machine based on stereolithography. The SLA used a laser to polymerize resin layers in 1-mm cross-sections, forming the surgical guide with cylindrical sockets for implants. After removing resin supports, a stainless-steel tube was inserted to ensure accurate implant angulation and positioning. The guide (In2Guide™, Cybermed, South Korea) was universally adaptable to any implant system.

The surgical template was cold sterilized and the drill box was autoclaved. Drilling began with a 2- mm drill and guide sleeve, progressing through 2.5-mm and 3-mm drills. The final drill was used freehand in an open universal guide system, following the osteotomy direction. Drilling was done at 800 rpm and the implant fixture was torqued to 30 Ncm.

**Fig. 1:**
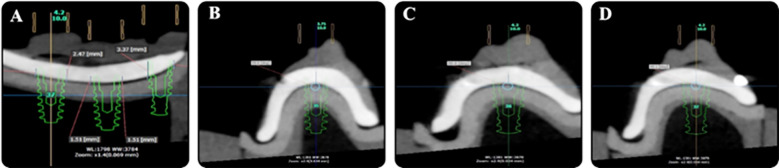
(A) Linear measurements (B) Angular measurements in #35, C: Angular measurements in #36, D: Angular measurements in #37

Post in-vitro implant placement with the aid of a universal open guide system, the implants placed in the replaceable bone blocks were tested for their positional accuracy using VMM (Fig.1). The measurements that were recorded were cross-referenced with the planned positions of implants placed virtually by CBCT. When determining the measurements for vertical distances between the implants, we calculated the distance between each implant and bone crest to implant platform on both mesial or distal directions and compared these values to those obtained from CBCT. However, when measuring angulations for all three implants, a horizontal reference line was drawn parallel to the base of the model at 7.5 mm above its base. All three implants were simulated at 90º to this reference line. The measurement denotations of all sub-groups are mentioned below (Table-III).

**Table-III:** Measurement Denotations of Sub-groups.

Group	Measurement Denotations	Subgroups Denotations
A (Control Group) Measurements of virtually planned implants by CBCT only)	L = Linear Distance	L _I-II_ = Linear distance in mesiodistal direction between Implants I and II L _II-III_ = Linear distance in mesiodistal direction between Implants II and III L _I-III_ = Linear distance in mesiodistal direction between Implants I and III
B (Guided Implant Placement)	V = Vertical Distance	V_M I_ = Vertical distance of mesial surface of Implant I from the ridge crest V_D I_ = Vertical distance from the distal surface of Implant I to the ridge crest V_M II_ = Vertical distance of mesial surface of Implant II from the ridge crest V_D II_ = Vertical distance of distal surface of Implant II from the ridge crest V_M III_ = Vertical distance of mesial surface of Implant III from the ridge crest V_D III_ = Vertical distance of distal surface of Implant III from the ridge crest
	P = Angulation/ Perpendicularity	P _I_ = Angle between Implant I and horizontal reference line P _II_ = Angle between Implant II and horizontal reference line P _III_ = Angle between Implant III and horizontal reference line

The data were analyzed using SPSS (ver. 25) statistical software. Descriptive statistics were computed for all the variables of Group-B (Guided Implant placement) in comparison to the control group (CBCT) with regards to each of the parameters being studied, i.e., vertical distance, linear distance and perpendicularity. Additionally, paired sample t-test was employed to observe statistical difference between the groups. The *P* value of less than 0.05 was considered significant.

## RESULTS

Linear, vertical and perpendicular distance values measured between Group-A and Group-B at various locations are presented in Table-IV. The statistically significant differences in linear distances between the two groups at various locations were observed (*p*< 0.05). In contrast, Group-B demonstrated a statistically significant difference (*p*< 0.05) between the distal surface of Implant I and the implant crest while horizontal reference lines of all three implants showed significant differences between Groups A & B (*p*< 0.05). The comparison between Group-A and Group-B demonstrated statistically significant differences in linear, vertical and angular implant placement accuracy. In terms of linear accuracy, Group-B consistently showed shorter inter-implant distances than those planned in Group-A. Specifically, the distance between Implants I and II was reduced from 8.79 mm to 7.70 ± 0.58 mm (*p* = 0.001) and the distance between Implants II and III decreased from 8.71 mm to 8.11 ± 0.30 mm (*p* = 0.001). The cumulative distance between Implants I and III also showed a significant reduction from 17.50 mm to 15.80 ± 0.48 mm (*p*< 0.001).

**Table-IV:** Evaluation and comparison of linear and vertical distance and perpendicularity values obtained between implants planned virtually (Group-A) and those placed through a stereolithographic surgical open guide system (Group-B).

Linear, Vertical & Perpendicularity Distance Values	Mean ± Standard Deviation (mm)		Significance *P* – value (2 - tailed)
	Group-A	Group-B	
L _I-II_	8.79 ± 0	7.70 ± 0.58	0.001*
L _II-III_	8.71 ± 0	8.11 ± 0.30	0.001*
L _I-III_	17.50 ± 0	15.80 ± 0.48	0.000*
V _MI_	1.51 ± 0	1.37 ± 0.32	0.287
V _DI_	3.37 ± 0	1.86 ± 0.48	0.000*
V _M II_	1.51 ± 0	1.65 ± 0.48	0.432
V _D II_	1.51 ± 0	1.56 ± 0.23	0.552
V _M III_	2.47 ± 0	1.79 ± 0.36	0.001*
V _D III_	1.51 ± 0	1.29 ± 0.39	0.156
P _I_	90.00 ± 0	84.52 ± 5.43	0.025*
P _II_	90.00 ± 0	83.57 ± 1.52	0.000*
P _III_	90.00 ± 0	80.41 ± 2.37	0.000*

Vertical accuracy was more variable. While some measurements, such as the mesial aspect of Implant-I, were not statistically different (*p* = 0.287), significant discrepancies were noted at other sites. The distal aspect of Implant-I showed a marked reduction from 3.37 mm to 1.86 ± 0.48 mm (*p*< 0.001) and the mesial aspect of Implant-III decreased from 2.47 mm to 1.79 ± 0.36 mm (*p* = 0.001), indicating vertical under-insertion in several cases. Angular accuracy also revealed significant deviations from the planned 90º angulation. Implant I was placed at 84.52 ± 5.43º (*p* = 0.025), Implant II at 83.57 ± 1.52º (*p*< 0.001) and Implant III at 80.41 ± 2.37º (*p*< 0.001), all of which were significantly different from the ideal angle.

## DISCUSSION

This study demonstrated the high angular and linear accuracy of a stereolithographic open guide system when compared to CBCT-based planning. Our findings provide a deeper assessment into how open universal guides can offer better outcomes to more expensive static navigation systems. The critical role of optimal implant positioning and orientation has been consistently emphasized in previous studies.^14^ Biomechanical considerations underscore the vulnerability of dental implants to nonaxial forces, particularly shear forces that can lead to bone resorption and implant fatigue failure.^15^ The angulation of implants has been identified as a key factor influencing stress distribution, with parallel placement recommended to mitigate excessive forces and minimize the risk of complications such as crestal bone loss and implant displacement.^15^,^16^ The use of computer-guided (static) surgery has emerged as a modern and effective tool for enhancing accuracy in implant placement, allowing for precise three-dimensional visualization and preoperative planning.^16^,^17^

The results demonstrated significant differences in linear, angular and vertical accuracy between the methods. Compared to closed guide system, the universal open guide system showed superior mesiodistal linear accuracy for implants I-II and improved angular accuracy. However, the observed lower vertical accuracy can be attributed to variations in the drilling step in the open guide technique, elastic deformation of resin during insertion or micromovements of the stereolithographic guide under pressure and operator-dependent variations in tactile feedback.^12^ The open guide system is recommended for achieving accurate horizontal and perpendicular implant positioning in cases involving multiple implants.^18^ However, caution is advised when placing implants vertically using this method and further research is needed to evaluate its applications and limitations across diverse clinical settings.

The findings of this study align with existing literature evaluating the accuracy of computer-guided implant surgery.^18^ This study provides evidence for how computer-aided surgery can make dental implant insertion predictable, precise, minimally invasive and efficient; thereby improving esthetics, enhancing patient comfort and satisfaction as well as facilitating immediate loading.^19^ Future investigations should focus on integrating depth-limiting mechanisms such as fully guided sleeves and adjustable drill stops to optimize vertical accuracy and prevent insertion errors in open-guide implant systems. Enhancing guide stability, especially posteriorly and incorporating dynamic navigation or real-time intraoperative verification may further improve vertical control. Clinical studies assessing the effects of drilling protocols, bone density and operator experience on vertical accuracy are essential for technique optimization and standardization.

### Limitations:

While the study yielded promising results, it has limitations. It assessed the accuracy of virtually planned implant placement using a universal open guide system (In2Guide™ Surgical Template and VMM), but did not consider the influence of operator experience on placement accuracy in computer-guided implant surgery.^2^,^20^-^22^ Also, the study did not fully evaluate drilling sequence and guide-hole design as factors affecting accuracy in static computer-assisted implant surgery as has been supported by previous studies.^20^

## CONCLUSIONS

Notable angular and vertical deviations, particularly in distal regions, were observed. with the stereolithographic open guide system. Overall, the stereolithographic open guide system approach outperformed CBCT-based virtual plans in horizontal (mesiodistal) placement, where it showed high linear as well as angular accuracy. It also matched navigation systems and closed-ended guidance systems in precision. The stereolithographic open guide can provide precise horizontal placements, which makes this system suitable for cases with multiple implants.

### Authors’ Contribution:

**AAA:** Conception, literature search, revision and approval of final version, responsible for integrity of this study.

**MMA:** Supervision, critical review and analysis.

**AAK:** Methodology, writing original manuscript, data collection and data interpretation.

**AW:** Drafting, design, interpretation and critical review.

All authors have read and approved the final version.

## References

[1] Gao SS, Zhang YR, Zhu ZL, Yu HY (2012). Micromotions and combined damages at the dental implant/bone interface. Int J Oral Sci.

[2] Maló P, Rangert B, Nobre M (2003). “All-on-Four”immediate-function concept with Brånemark System^®^implants for completely edentulous mandibles:a retrospective clinical study. Clin Impl Dent Res.

[3] Yao CJ, Cao C, Bornstein MM, Mattheos N (2018). Patient-reported outcome measures of edentulous patients restored with implant-supported removable and fixed prostheses:a systematic review. Clin Oral Impl Res.

[4] Buser D, Martin W, Belser UC (2004). Optimizing esthetics for implant restorations in the anterior maxilla:anatomic and surgical considerations. Int J Oral Maxillofac Impl.

[5] Feine J, Abou-Ayash S, Al Mardini M, De Santana RB, Bjelke-Holtermann T, Bornstein MM (2018). Group 3 ITI consensus report:Patient-reported outcome measures associated with implant dentistry. Clin Oral Impl Res.

[6] Worthington P, Rubenstein J, Hatcher DC (2010). The role of cone-beam computed tomography in the planning and placement of implants. J Amer Dent Assoc.

[7] Marquez Bautista N, Meniz-García C, López-Carriches C, Sánchez-Labrador L, Cortés-Bretón Brinkmann J, Madrigal Martínez-Pereda C (2024). Accuracy of different systems of guided implant surgery and methods for quantification:a systematic review. Appl Sci.

[8] Gurrea J, Bruguera A (2014). Wax-up and mock-up. A guide for anterior periodontal and restorative treatments. Int J Esthet Dent.

[9] Sayed AJ, Mohsin SF, Garg KK, Agwan MA, Tareen SU, Alruthea MS (2025). Finite Element Analysis (FEA) for Influence of Variation in Dental Implant Dimensions (Length and Diameter) on Peri-implant Bone Stress/Strain Distribution:A Systematic Review. Pak J Med Sci.

[10] Abutaleb FA, Borg HS, EKhalifa ME, Bader RR, Allam MK (2017). The accuracy of implant positioning using bone supported versus mucosa supported surgical guide templates for implant assisted lower complete overdenture. Egyp Dent J.

[11] Bagheri A, Jin J (2019). Photopolymerization in 3D printing. ACS Appl Poly Mater.

[12] El Kholy K, Lazarin R, Janner SF, Faerber K, Buser R, Buser D (2019). Influence of surgical guide support and implant site location on accuracy of static Computer-Assisted Implant Surgery. Clin Oral Impl Res.

[13] Li Y, Liu ZS, Bai XM, Zhang B (2013). Investigation of the effects of graded models on the biomechanical behavior of a bone-dental implant system under osteoporotic conditions. Pak J Med Sci.

[14] Alresayes S, Mokeem SA, Alhenaki AM, Vohra F, Abduljabbar T (2021). Evaluation of the implant diameter on the initial-stability of narrow-and standard-diameter implants placed in simulated Type-I and Type-IV bone-blocks. Pak J Med Sci.

[15] Manea A, Bran S, Dinu C, Rotaru H, Barbur I, Crisan B (2019). Principles of biomechanics in oral implantology. Med Pharm Rep.

[16] D'haese J, Ackhurst J, Wismeijer D, De Bruyn H, Tahmaseb A (2017). Current state of the art of computer-guided implant surgery. Periodont 2000.

[17] Guentsch A, An H, Dentino AR (2022). Precision and trueness of computer-assisted implant placement using static surgical guides with open and closed sleeves:An in vitro analysis. Clin Oral Impl Res.

[18] Beretta M, Poli PP, Maiorana C (2014). Accuracy of computer-aided template-guided oral implant placement:a prospective clinical study. J Periodont Impl Sci.

[19] Schnitman PA, Hayashi C, Han RK (2014). Why guided when freehand is easier, quicker and less costly?. J Oral Impl.

[20] Rungcharassaeng K, Caruso JM, Kan JY, Schutyser F, Boumans T (2015). Accuracy of computer-guided surgery:a comparison of operator experience. J Prosthet Dent.

[21] Youssef H, Maged AR (2023). Rehabilitation of the edentulous maxilla with all-on-four hybrid prosthesis and bar-clip retained overdenture in patients with mandibular hybrid prostheses:clinical, radiographic and prosthetic outcomes. Egyp Dent J.

[22] Brandão TB, Vechiato-Filho AJ, Vedovato E, Silva LS, Dos Santos Silva AR, Brito e Dias R (2021). Is the Fixed Mandibular 3-Implant Retained Prosthesis Safe and Predicable for Full-Arch Mandibular Prostheses?A Systematic Review. J Prosthodont.

